# Molecular Characterization and Overexpression of *VpRPW8s* from *Vitis pseudoreticulata* Enhances Resistance to *Phytophthora capsici* in *Nicotiana benthamiana*

**DOI:** 10.3390/ijms19030839

**Published:** 2018-03-05

**Authors:** Gongti Lai, Peining Fu, Yunxiao Liu, Jiang Xiang, Jiang Lu

**Affiliations:** 1 College of Food Science and Nutritional Engineering, China Agricultural University, Beijing 100083, China; laigt4769@cau.edu.cn (G.L.); fupeining@cau.edu.cn (P.F.); yunxiao_liu@cau.edu.cn (Y.L.); 2 Center for Viticulture and Enology, School of Agriculture and Biology, Shanghai Jiao Tong University, Shanghai 200240, China; xiangjiang717@163.com; 3 Guangxi Crop Genetic Improvement and Biotechnology Laboratory, Guangxi Academy of Agricultural Sciences, Nanning 530007, China

**Keywords:** *Vitis pseudoreticulata*, *RPW8*, downy mildew, *Plasmopara viticola*, *Phytophthora capsici*, overexpression

## Abstract

*RPW8* genes are atypical broad-spectrum genes that provide resistance to powdery mildew, downy mildew, the cauliflower mosaic virus in *Arabidopsis thaliana*, and powdery mildew in tobacco. They play important roles in basal plant pathogen defense. They also provide insights into a novel disease resistance mechanism. In this study, we report on homologous *RPW8* genes in *Vitis pseudoreticulata.* Five *VpRPW8* genes were cloned; their Open Reading Frame (ORF) sequences ranged from 1994 base pairs to 2478 base pairs. They were comprised of five exons and four introns and shared 78.66% identity. Their proteins had typical conserved RPW8 and NB-LRR (the nucleotide-binding site and the leucine-rich repeats) domains (except VpRPW8-d, which lacked LRR domains). Prokaryotic expression results were consistent with predicted molecular weights. All five *RPW8* genes were located in the cytoplasm. Quantitative real-time PCR (qRT-PCR) analysis showed that *VpRPW8s* in *V. pseudoreticulata* were induced by *Plasmopara viticola*, but nearly only *VvRPW8-d* genes were induced in *Vitis vinifera.* Furthermore, a *VpRPW8* transgenic tobacco system was established. Overexpressed *VpRPW8s* enhanced resistance to *Phytophthora capsici* and *VpRPW8s* conferred varying degrees of resistance to *Ph. capsici* in *Nicotiana benthamiana*. Our study presents novel members of the plant *RPW8* family and suggests that *VpRPW8s* are involved in enhanced resistance to *P. viticola* and *Ph. capsici*.

## 1. Introduction

Grapevine (*Vitis vinifera*) is the fourth flowering plant, the second woody plant, and the first fruit crop whose genome has been completely sequenced [1]. Grapevine contends with real disease threats such as downy mildew (DM), powdery mildew, and anthracnose. Downy mildew, caused by *Plasmopara viticola* (Pv), is one of the most serious diseases in grapevines. *P. viticola* is responsible for substantial yield losses in grapevines. *P. viticola* is a biotrophic oomycete pathogen germinating from zoospores. Its hyphal tips penetrate stomatal openings and develop mycelia with haustoria inside the mesophyll tissue. A new round of infection occurs when sporangia form and release sporangiophores after the initial infection. *P. viticola* is an obligate grapevine parasite. It can severely damage *V. vinifera* leaves, young shoots, tendrils, and fruits [2,3,4,5]. However, there are many wild grapevine germplasms which are resistant to this disease in China [6,7,8]. *V. pseudoreticulata*, a Chinese endemic wild grapevine, is highly resistant to several diseases including downy mildew [9]. *V. pseudoreticulata* has colonized a wide range of habitats and soils, and harbors many predicted resistance genes (*R* genes). *R* gene cloning, validation, and application have been important grapevine research objectives. Over the past several years, more than 13 loci resistant to *P. viticola* (*rpv*) have been mapped on various chromosomes in *Muscadinia rotundifolia*, north American cultivars, and *V. amurensis* [10,11,12,13,14,15,16,17]. Only *rpv1* was identified in a cross between *V. vinifera* ‘Syrah’ and *M. rotundifolia* “28-8-78” [10]. Nevertheless, there have been few studies on quantitative trait loci (qtl) or resistance genes in *V. pseudoreticulata*. 

Plants and pathogens have evolved sophisticated relationships. Basal defense and *R* gene-mediated defense are the two major types of disease resistance in plants [18]. Basal defense in plants includes innate immunity involved in pattern-triggered immunity (PTI) [19,20,21]. *R* gene-mediated defense is plant-adaptive immunity involved in effector-triggered immunity (ETI) [22]. Once a pathogen secretes effectors to breach basal defense, plants activate *R* genes that recognize the effectors and prevent pathogen invasion. In cloned *R* genes, the nucleotide-binding site (NB) domains and the leucine-rich repeats (LRR) constitute the majority. In *Arabidopsis thaliana*, resistance to powdery mildew8 (*RPW8*) consists of *RPW8.1* and *RPW8.2* genes. These genes were first characterized to be resistant to many powdery mildew pathogens. As atypical *R* genes, *RPW8.1* and *RPW8.2* had no NB or LRR domains but were able to induce localized, salicylic acid (SA)-dependent defenses [23]. When powdery mildew pathogens form haustoria to absorb plant nutrients, *RPW8.2* is induced and targets extra-haustorial membranes (EHM). Utilizing the SA signaling pathway, *RPW8.2* induces haustorial complex formation and hydrogen peroxide (H_2_O_2_) accumulation. Therefore, haustorial interception is the key to reducing oxidative damage in the host cell for *RPW8-*mediated mildew resistance [23,24,25].

Overexpressing *RPW8* genes enhance resistance to powdery mildew, *Hyaloperonospora parasitica* and the cauliflower mosaic virus [26]. Studies have demonstrated that *RPW8* genes enhance basal defense against biotrophic pathogens, which varies from powdery mildew-specific *R* genes. Based on this hypothesis, we conducted experiments to verify the disease resistance functions of *VpRPW8s*. In this study, we isolated five *RPW8* genes from *V. pseudoreticulata* and compared their transcriptional expression patterns with those in *V. vinifera* in response to *P. viticola* infection. We also assessed *Phytophthora capsici* resistance in transient transgenic tobacco overexpressing *VpRPW8* genes. Finally, we generated stable transgenic tobacco lines overexpressing *VpRPW8* genes in order to elucidate *VpRPW8s* resistance functions.

## 2. Results

### 2.1. Isolation, Characterization, and Bioinformatics of VpRPW8s

**Figure 1:**
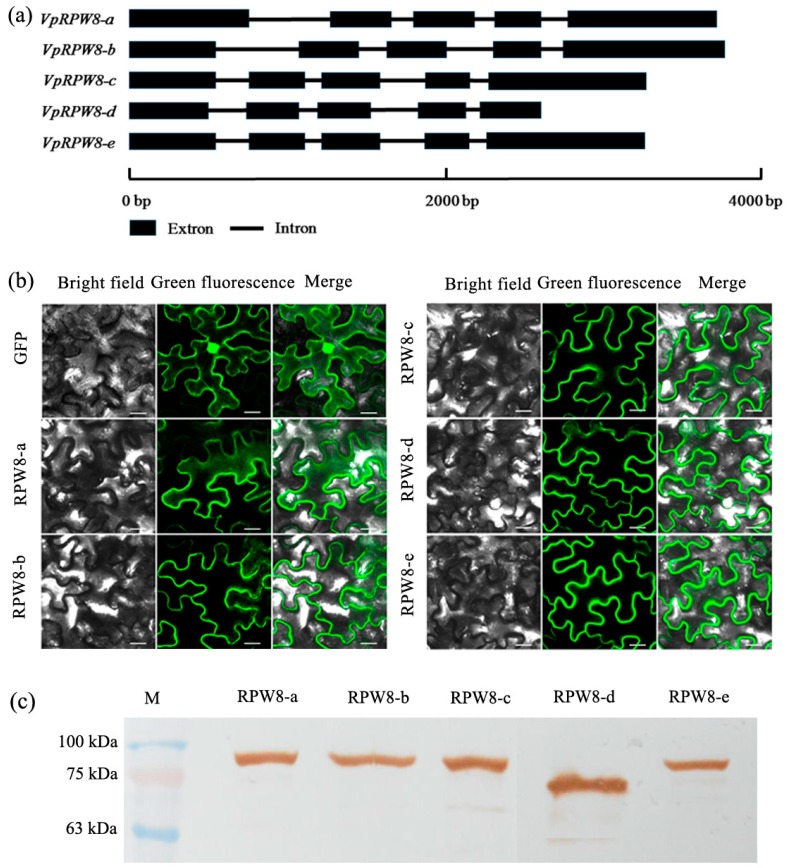
Gene structure, subcellular location, and prokaryotic expression of *VpRPW8s*. (**a**) Gene structure of *VpRPW8s* consisted of five exons and four introns. They showed different gene length, but similar exon and intron structure. They can be grouped into two different gene structure models based on their different intron length. (**b**) Subcellular location of *VpRPW8s*. *VpRPW8s* were localized in the cytoplasm. Scale bar = 20 μm. (**c)** Prokaryotic expressions of *VpRPW8s*. VpRPW8-a, VpRPW8-b, VpRPW8-c, and VpRPW8-e were between 75 and 100 kD whereas VpRPW8-d was ~75 kD. The results of prokaryotic expression were consistent with predicted data.

Five *RPW8* genes were obtained from *V. pseudoreticulata.* These genes were named *VpRPW8-a* (GenBank: KU365990), *VpRPW8-b* (GenBank: KU365991), *VpRPW8-c* (GenBank: KU365992), *VpRPW8-d* (GenBank: KX389173), and *VpRPW8-e* (GenBank: KX389175). *RPW8-d* had the shortest ORF (1994 base pairs) and *RPW8-a* had the longest (2478 base pairs). The average ORF length was 2358 base pairs. The genes encoded proteins 647 to 825 amino acid residues long. The average protein product molecular weight was 89.11 kD. Multiple gene sequence alignments showed that they share an average of 78.66% identity. *RPW8-a* and *RPW8-d* shared the lowest identity (37.33%) whereas *RPW8-c* and *RPW8-e* shared the highest identity (98.98%) (see Supplementary Table S1). Gene structure analysis revealed that all five genes contained five exons and four introns (see Figure 1a). *RPW8-a* and *RPW8-b* had long introns and shared the same gene structure model. The sizes of the four introns in *RPW8-c*, *RPW8-d*, and *RPW8-e* were relatively shorter and more uniform. Therefore, *RPW8-c*, *RPW8-d*, and *RPW8-e* were grouped in another gene structure model. Additional information on *VpRPW8s* can be found in Supplementary Table S2.

**Figure 2:**
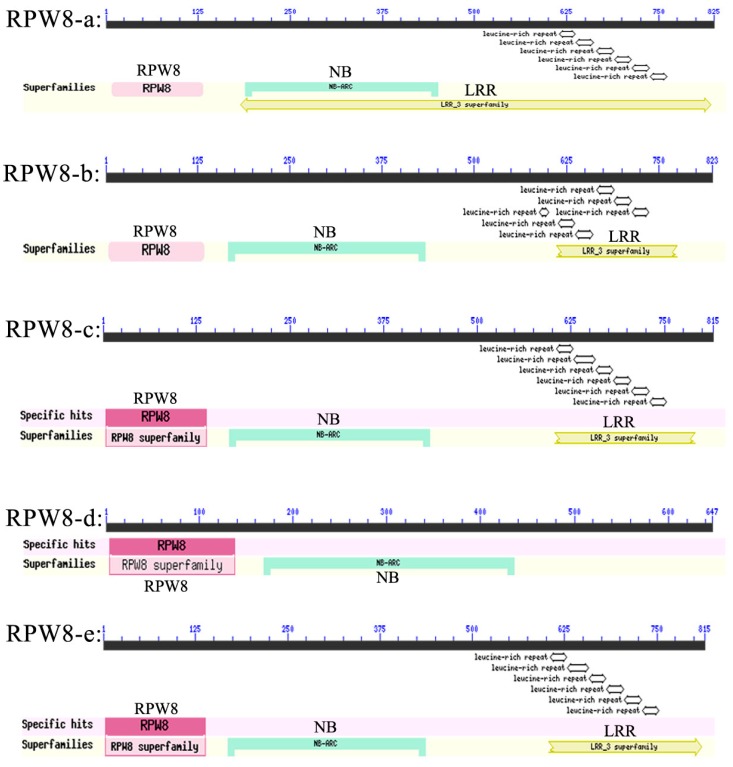
Conserved domains analysis of VpRPW8s. RPW8s contained RPW8 domains, conserved disease resistance domains NB (nucleotide binding sites) and LRR (leucine-rich repeats) except for VpRPW8-d. VpRPW8-d had the RPW8 and NB domains but not LRR. VpRPW8-a had a long LRRs motif starting from ~AA residue 183 and extending to ~AA residue 821. The other LRR motifs started from ~AA residue 600 and extended to ~AA residue 800.

Conserved domain predictions showed that they all had RPW8 and disease resistance domains like NB (nucleotide binding site) and LRR (leucine-rich repeat) (Figure 2). The exception was *VpRPW8-d*, which lacked LRR. VpRPW8-a had a long LRR motif starting from ~ amino acid (AA) residue 183 and extending to ~AA residue 821. The other LRR motifs started from ~AA residue 600 and extended to ~AA residue 800. Previous studies showed that the RPW8 motif was the atypical *R* gene domain and the NB-LRR motif was the typical *R* gene domain. As a result, VpRPW8 proteins had both the atypical RPW8 broad-spectrum structure and the typical *R* gene structure. Therefore, the *VpRPW8s* may be defined as functional disease resistance genes.

### 2.2. Transcriptional Expression Analysis of the RPW8s in V. pseudoreticulata and V. vinifera under P. viticola Stress 

**Figure 3:**
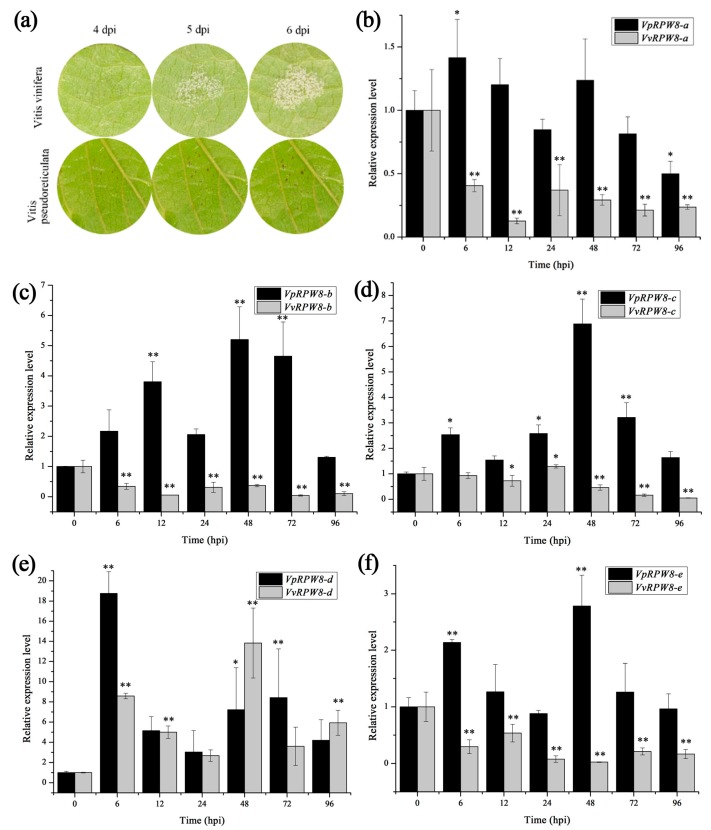
Transcriptional expression of *RPW8s* in *V. pseudoreticulata* and *V. vinifera* in response to *P. viticola*. (**a**) The disease symptom of *V. vinifera* and *V. pseudoreticulata* under *P. viticola* infection. (**b**–**f**) Transcriptional expression of *RPW8s* in *V. pseudoreticulata* and *V. vinifera* in response to *P. viticola*. *RPW8* genes were significantly upregulated in *V. pseudoreticulata* but downregulated in *V. vinifera* (RPW8-a, b, e). In *V. vinifera*, the expression levels were lower than those for the control (0 hpi) in all treatments except for *VvRPW8-d* (all treatments) and *VvRPW8-c* (24 hpi). *VvRPW8-d* genes were upregulated and had high expression levels at 6 and 48 hpi. The transcriptional levels of *VpRPW8s* changed significantly in the early stages of pathogenesis, reached a maximum, and then gradually decreased. The exception was *VpRPW8-a*, which was not significantly upregulated (except for 6 hpi, ** *p* < 0.01, * *p* < 0.05).

*V. pseudoreticulata* has resistant compounds whereas *V. vinifera* is susceptible. Nevertheless, little is known about the *R* genes in grapevines or how they work. Whether the *RPW8s* play important roles in *P. viticola* infection remains unknown. In this study, the transcriptional expression levels of the five *RPW8* genes were determined under *P. viticola* stress. The results showed that the *RPW8s* expressed differently in *V. pseudoreticulata* and *V. vinifera* (see Figure 3). The *RPW8* genes were abundantly upregulated in *V. pseudoreticulata* but their expression levels varied somewhat in *V. vinifera.* In *V. vinifera*, the expression levels were lower than those for the control (0 hpi) in all treatments except for *VvRPW8-d* (all treatments) and *VvRPW8-c* (24 hpi). *VvRPW8-d* genes were upregulated and had high expression levels at 6 and 48 hpi. The transcription levels of *VpRPW8s* changed significantly in the early stages of pathogenesis, then reached a maximum, and gradually decreased. The exception was *RPW8-a*, which was not dramatic upregulated. Taken together, all *VpRPW8s* were induced by *P. viticola* in *V. pseudoreticulata*, rather than *VvRPW8s*. Therefore, *VpRPW8s* participated in downy mildew resistance rather than *VvRPW8s*.

Two different expression patterns were detected. The first was early (6 hpi) induction followed by sustained high expression levels at 48 hpi. This pattern applied to *VpRPW8-a*, *VpRPW8-d*, and *VpRPW8-e.* In the second pattern, induction gradually increased and reached a maximum at 48 hpi. Such was the case for *VpRPW8-b* and *VpRPW8-c.* A wide range of expression efficiency was observed for *VpRPW8s. VpRPW8-d* genes were the most strongly induced and had an 18.7× expression level at 6 hpi. The expression levels of *VpRPW8-b* and *VpRPW8-c* peaked at 48 hpi with 5.2× and 6.8×, respectively. *VpRPW8-a* and *VpRPW8-e* genes were expressed at far lower levels, which were 1.4× (6 hpi) and 2.8× (48 hpi), respectively. Therefore, *VpRPW8s* (*RPW8-d* and *RPW8-c* as well as *RPW8-b*, *RPW8-e*, and *RPW8-a* in sequence) play key roles in response to *P. viticola* infection in *V. pseudoreticulata.* In contrast *VvRPW8s* (except *VvRPW8-d*) may not be actively involved in the defense against *P. viticola* in *V. vinifera.*

### 2.3. Prokaryotic Expression and Subcellular Localization of VpRPW8s

In this study, *pET30a-RPW8s-His* were constructed and transformed into *E. coli BL21*. Prokaryotic *VpRPW8s* expressions were examined by using Western blot. The results indicated that the VpRPW8-a, VpRPW8-b, VpRPW8-c, and VpRPW8-e proteins ranged in size from 75 to 100 kD. VpRPW8-d was ~75 kD (see Figure 1c). A bioinformatics analysis showed that the RPW8 proteins were 93.92, 93.04, 92.45, 73.62, and 92.52 kD, respectively. Therefore, the prokaryotic expression was consistent with predicted data and the *VpRPW8s* expressed their protein products as expected. 

To determine the subcellular locations of the *VpRPW8s*, *pBI121-RPW8s-GFP* were constructed and transformed into *Agrobacterium tumefaciens*. The *A. tumefaciens* suspensions were injected into *Nicotiana benthamiana* leaves to express the RPW8s-GFP fusion proteins. GFP fluorescent signals for the five *RPW8* genes were observed by using confocal microscopy and found mainly in the cytoplasm (see Figure 1b), which suggests the cytoplasmic location for these five *VpRPW8* genes.

### 2.4. Transiently Expressing RPW8s Enhanced Resistance to Ph. capsici 

**Figure 4:**
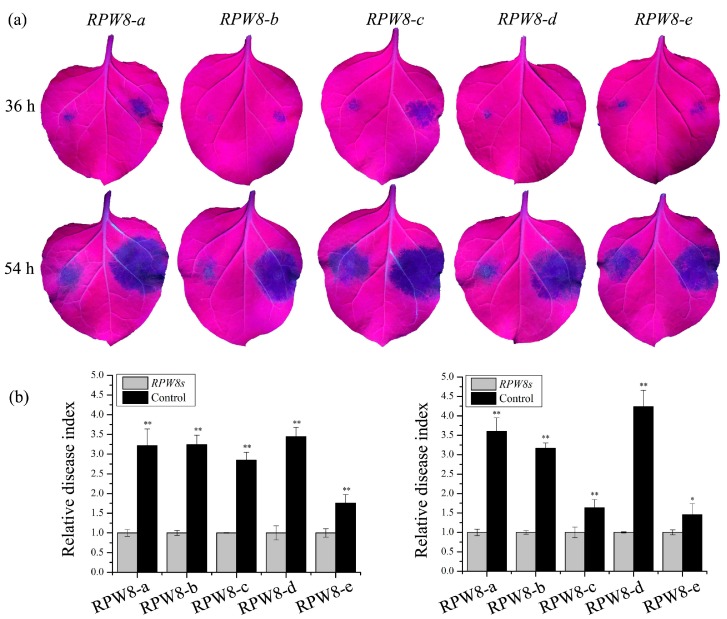
Transiently expressing *VpRPW8s* enhanced *Ph. capsici* resistance in *N. benthamiana*. (**a**) Lesions on the *N. benthamiana* leaves transiently expressing *VpRPW8s* at 36 hpi and 54 hpi. Symptoms on the right side of the leaves represent the control while those on the left side represent transiently expressing *VpRPW8s*. The necrotic areas expanded over time (36–54 hpi). The experimental group showed somewhat smaller necrotic areas. (**b**) Disease indices for *N. benthamiana* at 36 hpi. (**c**) Disease indices for *N. benthamiana* at 54 hpi. Disease index (Y-axis) is the ratio of the control necrotic areas to those on the transgenic plants (** *p* < 0.01, * *p* < 0.05).

To determine whether *VpRPW8s* enhance *Ph. capsici* resistance in *N. benthamiana*, transiently transgenic tobacco leaves were inoculated with *Ph. capsici*. As shown in Figure 4a, there were signs of necrosis in the tobacco leaves at 36 hpi. Necrosis was more severe in the controls on the right side of the leaves than the experimental group expressing *VpRPW8s* on the left side of the leaves. At 54 hpi, the necrotic areas expanded and the difference between the control and the experimental groups was more significant. 

The resistance to *Ph. capsici* induced by transient *RPW8s* expression was investigated by calculating the areas of leaf necrosis in *N. benthamiana.* The ratio of control to transgenic leaf necrotic areas was defined as the relative disease index. The relative disease index increased when disease symptoms grew. Statistical analysis showed that the relative disease index of controls was significantly higher than in *VpRPW8* transgenic groups (see Figure 4b,c). Therefore, *VpRPW8s* enhanced *Ph. capsici* resistance in transiently transformed *N. benthamiana*. 

### 2.5. Stably Expressing RPW8s Enhanced Resistance to Ph. capsici 

**Figure 5:**
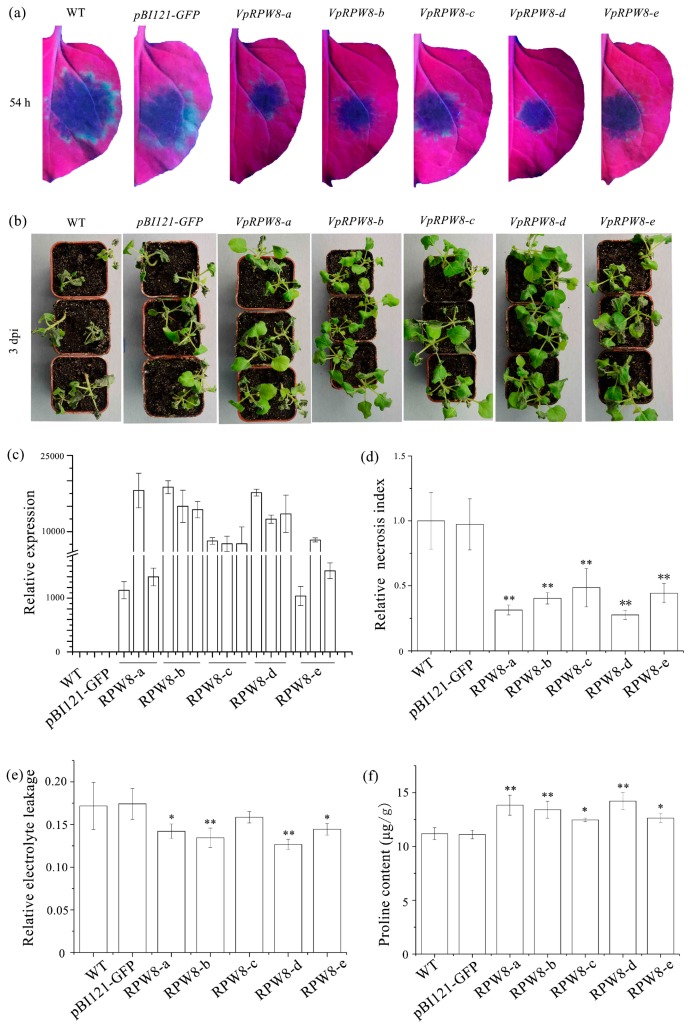
*VpRPW8s* enhanced *Ph. capsici* resistance in transgenic *N. benthamiana*. (**a**) Lesions on detached *N. benthamiana* leaves expressing *VpRPW8s*. (**b**) Disease symptoms in whole *N. benthamiana* plants expressing *VpRPW8s*. (**c**) Transcriptional expression of *RPW8*s in transgenic *N. benthamiana* (T2). (**d**) Relative disease index on detached *N. benthamiana* leaves expressing *VpRPW8s*. (**e**) Relative electrolyte leakage in whole *N. benthamiana* plants expressing *VpRPW8s*. (**f**) Proline content in whole *N. benthamiana* plants expressing *VpRPW8s* (** *p* < 0.01, * *p* < 0.05).

The aforementioned experiments revealed specific *RPW8s* structures and demonstrated their disease resistance functions. Based on the results of the transient *VpRPW8s* transformation experiment, stable transformations were conducted. All five transgenic lines were obtained by kanamycin resistance selection (T1) and by using DNA-PCR detection (T1). More than Forty-five T2 seedlings were then germinated from T1 seeds in kanamycin resistance medium. Transcriptional analysis of these T2 lines indicated that more than 45 plants or all of the plants studied overexpressed *RPW8s* (see Figure 5c). 

To verify *Ph. capsici* resistance, we inoculated detached leaves and living plants with the pathogen (see Figure 5a). Necrotic areas (relative disease index) on the detached leaves in order of increasing size were labeled *RPW8-d*, *RPW8-a*, *RPW8-b*, *RPW8-e*, *RPW8-c*, and CK (WT and *pBI121-GFP* in Figure 5d). These results somewhat corroborated the data obtained for the transient transformation experiment. This showed the transgenic group was more resistant to *Ph. capsici* than the control groups. 

Both the control and transgenic plants displayed varying degrees of disease symptoms. As shown in Figure 5b, the control plants were severely wilted while a few turgid leaves remained in the plants of the experimental group. Since the previous experiments indicated that *P. viticola* significantly induced *VpRPW8s* at 48 hpi, we measured relative electrolyte leakage and proline content at 48 hpi to determine the disease resistance response. The relative electrolyte leakage in the *VpRPW8* transgenic plants was lower than that in the control plants (see Figure 5e). In contrast, the proline content in the *VpRPW8* transgenic plants was higher than that in control plants (see Figure 5f). These findings indicate that *RPW8s* enhanced *Ph. capsici* resistance in *N. benthamiana*.

The relative electrolyte leakages in order of increasing magnitude were labeled as *RPW8-d*, *RPW8-a*, *RPW8-b*, *RPW8-e*, *RPW8-c* and CK (WT and *pBI121-GFP*). The proline contents in order of decreasing magnitude were labeled as *RPW8-d*, *RPW8-a*, *RPW8-b*, *RPW8-e*, *RPW8-c*, and CK (WT and *pBI121-GFP*). Therefore, *N. benthamiana* plants expressing *VpRPW8s* were more resistant to *Ph. capsici* than CK plants. *VpRPW8-d* genes were deduced to be the most resistant followed by *VpRPW8-a*, *VpRPW8-b*, *VpRPW8-e* and *VpRPW8-c* in sequence.

## 3. Discussion

### 3.1. Conserved Domains and Sequence Polymorphism of RPW8s

In plants, the *RPW8* genes constitute a superfamily. They were first reported in *A. thaliana* in 2001 [23]. It was already known that the *RPW8s* had an atypical *R* gene structure but the same mechanism as *R* genes. All *RPW8s* share the conserved RPW8 domains. The predicted *AtRPW8.1* and *AtRPW8.2* genes have 45.2% sequence identity [23]. In *Brassica spp.*, they are highly homologous and their sequence identity ranges from 90% to 95% [27,28]. In the present study, we predicted the typical R gene domain NB-LRR as well as the conserved RPW8 domains. Multiple sequence alignments of *RPW8* genes showed 78.66% nucleotide and 74.28% protein identity. This seemed an apparently high identity, but pairwise sequence alignment revealed that the nucleotide sequence identity ranged from 37.33% (protein, 25.45%) to 98.98% (protein, 97.67%) (see Supplementary Table S1). 

*The AtRPW8.1* gene was localized in the mesophyll cells and was surrounded by punctuated spots proximal to the chloroplasts. In contrast, the *AtRPW8.2* gene was observed in both the epidermal and mesophyll cells. *AtRPW8.1* and *AtRPW8.2* genes were heterogeneous and may have spatially differed from each other. Nevertheless, they were both resistant to biotrophic pathogens [25,26]. In *V. pseudoreticulata*, *RPW8s* genes were localized mainly in the cytoplasm. Plant *RPW8s* are also diverse in terms of subcellular location. Therefore, *RPW8s* have conserved structures while also having sequence and functional polymorphism.

### 3.2. VpRPW8s Participate in Resistance to P. viticola

Previous studies indicated that *V. vinifera* was generally susceptible to downy mildew caused by *P. viticola*. In recent years, *Rpv1*, *NPR1* homologs, and PR protein-encoding genes have been identified as downy mildew resistance-associated genes [10,29,30]. In our previous study, we presented a series of candidate genes that may contribute to downy mildew resistance [31]. To date, however, little is known about *R* genes, and obstacles persist in *V. vinifera* breeding improvements. In contrast, the Chinese wild grape *V. pseudoreticulata* showed strong resistance to downy mildew. For this reason, *V. pseudoreticulata* was used as a germplasm to breed disease resistance and has proven to be an invaluable entity in functional genomics. In our study, *RPW8s* are differentially expressed in *V. vinifera* and *V. pseudoreticulata* in response to *P. viticola*. *VpRPW8 genes* were strongly induced by *P. viticola*, which first rose and then fell in expression levels over time. Two different expression patterns were observed for *VpRPW8 genes*. In the first pattern, *VpRPW8-a*, *VpRPW8-d*, and *VpRPW8-e* were involved, which indicates that *RPW8s* were induced early and remained at a high expression level during the middle stage. In the second pattern, *VpRPW8-b* and *VpRPW8-c* were significantly induced and reached a peak during the middle stage. On the other hand, the expression levels of *VvRPW8s* (except *VvRPW8-d*) remained low and did not respond strongly to *P. viticola*. The expressions of the *VpRPW8 genes* resembled those for the *NB-LRR R* genes expressed at low levels prior to pathogen invasion, but were then rapidly induced during the early stage of infection [32,33,34,35]. Therefore, *VpRPW8s* are able to resist *P. viticola* in *V. pseudoreticulata.*

### 3.3. Broad-Spectrum Disease Resistance of RPW8

A study of the range of pathogens controlled by *RPW8* in *A. thaliana* showed that transgenic plants were resistant to all powdery mildew pathogens tested [23]. *A. thaliana* expressing *RPW8* genes also had enhanced resistance to *Hyaloperonospora parasitica* and even appeared to be resistant to the cauliflower mosaic virus when expressing *RPW8s* from their native promoters [26]. *AtRPW8.1* and *AtRPW8.2* genes also induced resistance to powdery mildews in tobacco [36]. In our study, *P. viticola* induced *VpRPW8s* in *V. pseudoreticulata. VpRPW8s* played an important role in the response to *P. viticola* invasion. To examine *VpRPW8s* disease resistance further, we established a *VpRPW8s* overexpression system in *N. benthamiana* using *Agrobacterium*-mediated transformation. The excised leaves from both transiently and stably transformed transgenic plants infected with *Ph. capsici* had smaller necrotic areas than those of either the wild type or the control plants. Whole tobacco plants overexpressing *VpRPW8* genes appeared to be more resistant to *Ph. capsici* than either the wild type or the control. *VpRPW8* genes also enhanced resistance to *Phytophthora parasitica* in our other experiments. In summary, *VpRPW8s* enhanced resistance to *P. viticola*, *Ph. capsici*, and *Ph. parasitica*. Therefore, *VpRPW8s* are broad-spectrum disease resistant genes. 

## 4. Materials and Methods

### 4.1. Plant Materials and Treatments

One-year-old *V. pseudoreticulata* “1058” and *V. vinifera* “Cabernet Sauvignon” were grown in a greenhouse at 25 ± 2 °C under a 16 h light/8 h dark photoperiod. For quantitative real-time PCR, grapevine leaves were inoculated with 10 μL of an aqueous suspension of pathogen sporangia (10^5^ sporangia mL^−1^) and sampled at 0 hpi (hours post-inoculation), 6, 12, 24, 48, 72, and 96 hpi. The excised leaves were stored at −80 °C. One-month-old *N. benthamiana* seedlings in vivo were used for transient transformation and disease resistance evaluation. The third to fifth unfolded leaves from the shoot apex were excised for inoculation with *Ph. capsici*. One-month-old *N. benthamiana* seedlings in vitro were used for stable genetic transformation with *VpRPW8s*. Transgenic *N. benthamiana* seedlings were transplanted to pots in the greenhouse when they were one month old (4 to 6-leaf stage). 

### 4.2. Gene Cloning and Sequence Analysis

Total RNA was isolated from grapevine leaves according to a slightly modified cetyltrimethylammonium bromide (CTAB) method. The total RNA was used as a template for reverse transcription with a RevertAid TM First Strand cDNA Synthesis Kit (Invitrogen, Carlsbad, CA, USA). Primers for gene cloning were designed according to the five predicted *RPW8* sequences in Phytozome (https://phytozome.jgi.doe.gov/pz/portal.html#!search?show=KEYWORD) and PRG (http://prgdb.crg.eu/wiki/Main_Page). Primer data is listed in Supplementary Table S3. The PCR products were sub-cloned in *E. coli* and sequenced. Bioinformatics analysis of the five *RPW8s* was performed using DNAMAN, ProtParam tool (http://web.expasy.org/protparam/), and NCBI-CDD (http://www.ncbi.nlm.nih.gov/Structure/cdd/wrpsb.cgi).

### 4.3. Quantitative Real-Time PCR Analysis

To determine the transcription levels of the five *RPW8s* during the infections of *V. pseudoreticulata* and *V. vinifera* with *P. viticola*, leaves were excised and used for RNA extraction. The qRT-PCR was performed on the QIAGEN Rotor-Gene Q system (QIAGEN, Hilden, Germany) using a SuperReal PreMix Plus (SYBR Green) kit (TIANGEN Biotech Co.， Ltd., Beijing, China). To calculate the transcription levels, constitutively expressed elongation factor1-α (*EF1-α*) was used as an internal gene for normalization [37] with the 2^−∆∆*C*t^ method [38]. All reactions were performed in triplicate. 

### 4.4. Prokaryotic Expression and Subcellular Localization Analysis

For prokaryotic expression, the *RPW8s* PCR fragments were sub-cloned into *pET30a* to generate *pET30a-RPW8-a*, *pET30a-RPW8-b*, *pET30a-RPW8-c*, *pET30a-RPW8-d*, and *pET30a-RPW8-e*. The recombinant plasmids were then chemically transformed into *E. coli BL21* which was grown in Luria-Bertani (LB) broth supplemented with kanamycin until the OD_600_ reached 0.4. Then 10 mmol L^−1^ isopropyl β-d-1-thiogalactopyranoside (IPTG) was added to induce target protein expression. The proteins were verified by Western blot and dyed with 3′-diaminobenzidine (DAB).

For subcellular localization, *pBI121-RPW8-a-GFP*, *pBI121-RPW8-b-GFP*, *pBI121-RPW8-c-GFP*, *pBI121-RPW8-d-GFP*, and *pBI121-RPW8-e-GFP* were constructed using a *pBI121-GFP* plasmid (*CaMV 35S* promoter-driven). The recombinant plasmids were transferred via electroporation into the *Agrobacterium tumefaciens* strain *GV3101*. The *GV3101* containing these vectors was harvested by using centrifugation when the OD_600_ reached ~1.0 and resuspended in 10 mM MgCl_2_ to a final OD_600_ of 0.6. The transient transformations were performed according to a protocol previously described for *N. benthamiana* [39]. Fluorescence was visualized at 488 nm with an Olympus FluoView^TM^ FV1000 (Olympus Corporation, Shinjuku, Tokyo, Japan). 

### 4.5. Transient Transformation and Resistance to Ph. capsici in N. benthamiana

To determine *RPW8s* resistance to *Ph. capsici*, the aforementioned *A. tumefaciens* harboring *pBI121-RPW8s-GFP* were used to transform *N. benthamiana.* A 10 mM MgCl_2_ solution was prepared for transient transformation. Leaves were excised 48 h after being injected with the *A. tumefaciens* suspensions. *A. tumefaciens* harboring *pBI121-RPW8s-GFP* was injected on the left side of the back of the leaf while *pBI121-GFP* was injected on the right side as a type of control. *N. benthamiana* leaves were inoculated with *Ph. capsici* pathogen disks cultured on solid oat medium. Leaf lesions were photographed with UV imaging at 36 and 54 hpi. Disease index was analyzed according to the ratio of the control necrotic areas to those on the transgenic plants.

### 4.6. Stable Transformation and Resistance to Ph. capsici in N. benthamiana 

One-month-old in vitro *N. benthamiana* seedlings were used for genetic modification. The genetic manipulations were conducted as previously described, with minor modifications [40]. The bacterial strain and expressing vectors (*pBI121-RPW8s-GFP*) were described as transient expression, which was previously mentioned. To verify the transgenic *N. benthamiana* plants, kanamycin (60 mg L^−1^)-resistant selection and DNA-PCR were performed on T1 plants. Kanamycin-resistant selection and transcription level analysis were performed on T2 plants as well. The T2 plants were used to evaluate *VpRPW8s* disease resistance. Excised-leaf inoculation with *Ph. capsici* was performed as described above for the transient transformation. For the whole-plant inoculation, *Ph. capsici* zoospore suspensions were prepared and adjusted to 100 zoospores mL^−1^ [41]. *VpRPW8* plants were sprayed with zoospore suspensions. *N. benthamiana* wild type- and *pBI121-GFP* transgenic plants were used as controls. All plants were maintained at 25 ± 2 °C. The chambers were sealed with plastic film to retain moisture. Relative electrolyte leakage [42] and proline content [43] were measured as previously described. 

## 5. Conclusions

In this study, we cloned five *VpRPW8* genes from *V. pseudoreticulata* which is known to be highly resistant to downy mildew. These five genes harbor the same functional conserved domains as *RPW8* and *NB-LRR* genes except for the *VpRPW8-d* gene. All five *VpRPW8* genes consisted of five exons and four introns. Subcellular location showed they were localized in the cytoplasm. *VpRPW8s* were strongly induced by *P. viticola* in *V. pseudoreticulata*, rather than *VvRPW8s*.Therefore, *VpRPW8* genes participate in downy mildew resistance. *VpRPW8-d* was the most highly expressed gene, followed by *VpRPW8-b* and *VpRPW8-c. VpRPW8-a* and *VpRPW8-e* genes were expressed at low levels. Transient and stable transformations were carried out on *N. benthamiana* to observe the disease resistance functions of *RPW8s*. The results revealed that *VpRPW8* transgenic tobacco was highly resistant to *Ph. capsici*; *VpRPW8-d* was deduced to be the most resistant gene. Taken together, we verified the novel members of the plant *RPW8* family and enriched the R gene associated with the research in grapevines. *VpRPW8* genes possess conserved domains, and sequence and function polymorphism. These are also considered broad-spectrum disease genes. The study of broad-spectrum-like resistance genes lays the foundation for future research and application in grapevine variety improvement. 

## Supplementary Materials

The following are available online at http://www.mdpi.com/1422-0067/19/3/839/s1.

## Abbreviations

RPW8	Resistance to Powdery Mildew8
R gene	Resistance Gene
NCBI	National Center for Biotechnology Information Search database
qRT-PCR	Quantitative Real-Time PCR
GFP	Green Fluorescent Protein
ORF	Open Reading Frame
hpi	Hours Post-Inoculation
dpi	Days Post-Inoculation
