# Supplementary material for: Molecular Characterization and Overexpression of VpRPW8s from Vitis pseudoreticulata Enhances Resistance to Phytophthora capsici in Nicotiana benthamiana
Source: Int J Mol Sci. 2018 Mar 5;19(3):839. doi: 10.3390/ijms19030839 (PMC5877700; doi:10.3390/ijms19030839)
Supplement: Supplementary file 1 [file ijms-19-00839-s001.pdf]

Supplementary Material

Molecular Characterization and Overexpression of *VpRPW8s* from *Vitis pseudoreticulata* Enhance Resistance to *Phytophthora capsici* in *Nicotiana benthamiana*

Gongti Lai [1], Peining Fu [1], Yunxiao Liu [1], Jiang Xiang [1] and Jiang Lu [1,2*]

[1] College of Food Science and Nutritional Engineering, China Agricultural University, Beijing 100083, China;

[2] Center for Viticulture and Enology, School of Agriculture and Biology, Shanghai Jiao Tong University, Shanghai 200240, China

Supplementary Table

Table S1: Gene and protein identity

Genes	*RPW8-a*	*RPW8-b*	*RPW8-c*	*RPW8-d*	*RPW8-e*
RPW8-a	——	47.02/31.85	46.99/33.13	37.33/25.45	47.33/33.[illegible]
RPW8-b	47.02/31.85	——	83.66/71.22	65.3/55.38	83.17/70.[illegible]
RPW8-c	46.99/33.13	83.66/71.22	——	79.04/78.90	98.98/97.[illegible]
RPW8-d	37.33/25.45	65.3/55.38	79.04/78.90	——	78.56/78.[illegible]
RPW8-e	47.33/33.37	83.17/70.50	98.98/97.67	78.56/78.04	——

Commented [M1]: Please confirm the change made by editor.

Commented [A2R1]: We have confirmed the change.

Commented [M3]: Please confirm the red color is necessary or not.

Commented [A4R3]: We think it is necessary.

Table S2: Gene and protein information

Genes	GenBank ID	ORF lenth (bp)	Number of exon	Number of intron	Number of AA	Molecular weight(D)	Theoretical pI	hydropathicity
VpRPW8-a	KU365990	2478	5	4	825	93917.3	6.01	-0.088
VpRPW8-b	KU365991	2472	5	4	823	93041.59	5.81	-0.179
VpRPW8-c	KU365992	2448	5	4	815	92447.1	5.76	-0.178
VpRPW8-d	KX389173	1994	5	4	647	73622.88	5.66	-0.25
VpRPW8-e	KX389175	2448	5	4	815	92520.32	5.76	-0.175

Table S3: Primer sequence

Primer name	Primer sequence	Vector or purpose
Va-F	ATGGCAGTAACCGATTTATTCC	gene clone
Va-R	CATACAGTAGAATGGAGGAGTAATCA	
Va-qpcr-F	CCATACCTCTTTCTGCTAACG	q-RT-PCR
Va-qpcr-R	TTTGGTTTCGTGGGACTC	

Va-pet-F	*GGGGTACC*ATGGCAGTAACCGATTTATTCC	pET30a
Va-pet-R	*CGGGATCC*GACTCCACCAGCCAGTCCAGATC	
Va-gfp-F	*CGGGATCC*ATGGCAGTAACCGATTTATTCC	pBI-121-GFP
Va-gfp-R	*GGGGTACC*CGCTCCACCAGCCAGTCCAGATC	
Vb-F	ATGGCTTTGGCGCTTGTTGGAGG	gene clone
Vb-R	CCAATGCTTACATGTACAGCTGCTTC	
Vb-qpcr-F	GACCTGCAAATTCCTGAAAC	q-RT-PCR
Vb-qpcr-R	AGTCAGTTCAACTGCGATAC	
Vb-pet-F	*GGGGTACC*ATGGCTTTGGCGCTTGTTGGAG	pET30a
Vb-pet-R	*CCGCTCGAG*TAGCAAATGCCAGTTGATAC	
Vb-gfp-F	*CGGGATCC*ATGGCTTTGGCGCTTGTTGGAG	pBI-121-GFP
Vb-gfp-R	*GGGGTACC*GTAGCAAATGCCAGTTGATAC	
Vc-F	ATGGCTTTGGAGCTTGTTGGAGG	gene clone
Vc-R	GAATATAATTAGGCGTCCGGTTCA	
Vc-qpcr-F	CTCGTCTTTATAACTAGGGCAG	q-RT-PCR
Vc-qpcr-R	CTCCAACAAGCTCCAAAGC	
Vc-pet-F	*GGGGTACC*ATGGCTTTGGAGCTTGTTGGAG	pET30a
Vc-pet-R	*CCGCTCGAG*AAGCCAATTCAAGCTGATAAT	
Vc-gfp-F	*CGGGATCC*ATGGCTTTGGAGCTTGTTGGAG	pBI-121-GFP
Vc-gfp-R	*GGGGTACC*AAGCCAATTCAAGCTGATAAT	
Vd-F	ATGGCTTTGGAGCTTGTTGGAG	gene clone
Vd-R	TTATTCAGTTGCAAAAGCTTGACC	
Vd-qpcr-F	TTATGGTTTCTGTGCTGCTG	q-RT-PCR
Vd-qpcr-R	TTATTCAGTTGCAAAAGCTTG	
Vd-pet-F	*GGGGTACC*ATGGCTTTGGAGCTTGTTGGAG	pET30a
Vd-pet-R	*CCGCTCGAG*TTCAGTTGCAAAAGCTTGACC	
Vd-gfp-F	*CGGGATCC*ATGGCTTTGGAGCTTGTTGGAG	pBI-121-GFP
Vd-gfp-R	*GGGGTACC*CGTTCAGTTGCAAAAGCTTGACC	
Ve-F	ATGTCTGCGGAGCTTGTTGGAG	gene clone
Ve-R	TTAAAGCCAATTCAAGTTGATA	
Ve-qpcr-F	AGAAGGGAGAAACCAACTCTG	q-RT-PCR
Ve-qpcr-R	TCCAACAAGCTCCGCAGA	
Ve-pet-F	*GGGGTACC*ATGGCTTTGGAGCTTGTTGGAG	pET30a
Ve-pet-R	*CCGCTCGAG*AAGCCAATTCAAGCTGATAAT	
Ve-gfp-F	*CGGGATCC*ATGTCTGCGGAGCTTGTTGGAG	pBI-121-GFP
Ve-gfp-R	*GGGGTACC*CGAAGCCAATTCAAGTTGATAAT	
V-EF1α-F	GAACTGGGTGCTTGATAGGC	q-RT-PCR
V-EF1α-R	ACCAAAAATATCCGGAGTAAAAGA	
Nb-EF1α-F	AGAGGCCCTCAGACAAAC	q-RT-PCR
Nb-EF1α-R	TAGGTCCAAAGGTCACAA	

Supplementary sequence

>VpRPW8-a ORF 1-2478
ATGGCAGTAACCGATTTATTCCCCGGTGAAATCGCCGCGGAGCTCCTGAAAATTCTAATATCTATATCAAG
AGAATCGTTTATGTGTAAATCCAGCGCAGAACAAATGGTAACGTATATACAACAAATCCTCCCCATAATCC
AAGAAATACAGTACTCGGGCGTGGAATTGTCGGAGTTGCGACAGTTTCAGATCGATCGCTTCGTGCATACT
CTCCGCGAGGGCCAAGAGCTCGCCAAAAAAGTCCAGGCCTGCGGCAGATGGAACGTGTACAGGAAGGTGCA
GTTGGCGAGAAAGATGGAGAAGCTAGAGAAGAACATACTCAGGTTCTTGAATGGCCCCCTGCAGGCCCATA
TTCTGGCGGACGTCCACCACATGCGGTTCGAGTCAGCAGAGAGGTTCGACCGGCTGGAATTTTCGGCTCGG
CGGCTCGAGGAGCAGCTTGGAGGCATGAAGATCGGGGTTGGTGGCGGAGGGCGGTTGGCGGAGGCAGTGAA
GCGAGGGGGGGAGGAGGAGGAGAGTTGCGAAGGTTTGACAAGTATGGGGGTTGGGATGGCTTTAGGGAAGA
AGAAGGTGAAGGAAATGCTTATTGATAGAGACGATCTGCGGGTTGTTGGGATTCATGGGATTGGTGGCAGT
GGCAAAACTACTGTTGCCAAGGAGATTTGCAGAGATGGAGAAGTCCGAAGCTACTTTGATGACAGGATTCT
GTTCCTGACTGTGTCGCAGTCCCCAAACGTGGAGCAGTTGAGGTCTCATATTTCGGAATATATTGAAGGGA
AGGACATGATTAAATCCCATGGTCCCATTCGACGATGGAAGTCACAATTTGAGAGGAGGATTGGAGTACGT
ACACTTGTGGTTCTGGATGATATCTGGTCACTCTCAGTGCTCGAGCACCTGATTTCCAGAATACCTGGATG
CAAAACCCTTGTTGTTTCACGGTTCAAATTCCCAACTGTCCTCAACCTCACCTATGAGCTGGAATTGCTGA
GAGAAGATGAAGCTATCTCCTTGTTCTGTCATTTTGCTTTTGGGCAGAAGTCCATACCTCTTTCTGCTAAC
GAGAATTTGGTCAAACAGGTTGTGCGCGAGTGTAAAGGGCTTCCTTTGGCTCTTAAAGTGATTGGAGCTTC
ACTTAGAGACCGGCCCCAAATGTTTTGGGCAAGTGCAGCAAGCAGGCTATCACAAGGGGAACCCATTTGTG
AGTCCCACGAAACCAAATTGCTTGAACGAATGGCAGTTGGTATTGCTGACCTGCCAAAAATGGTTAGAGAA
TGTTTCTTGGACTTGGGAGCCTTTCCAGAGGATAAGAAGATTCCTCTTGAAGTTCTAATCAACATCTGGGT
TGAGATCCATGATCTTTTTCAGGAGGATGCTTTTGCAGTTCTTTATGCGCTTGCAGAAAAGAATCTCCTTT
CCCTGGTGAATGATGCAAGAGCTGGGGACATATATAGCAGCTATTTTGAGATCTCTGCTTCTCAGCATGAT
GTTTTAAGAGATCTTGCCCTTTACATGAGCAAGCGTGAGGGCATAAATGATCGAAGGCGTTTGCTTATGCC
AAGGAGGGAAACAAGACTTCCAAAAGAATGGGAGAGGAATATGGACCAGCCATTTAATGCCCAAATTGTTT
CAATTCATACAGGTGAAATGGGAGAAACAGACTGGTTCCAGATGGACTTGCCCAAGGCTGAGGTTCTTATC
CTCAACTTCTCCTCAAGTGAATACTTCCTACCTCCCTTCATTGATCGGATGCCAAAGCTTCGAGCTTTAAT
ATTAATCAATTACAGTACCTCAACAGCGATTCTGAATAATGTAGAAGTATTTTCCAAATTAACTAACTTGA
GGAGCCTCTGGTTTGAGAAGATTTCTATCCCAGAGTTTCCCAGAACTACTATCCCCATGAAAAGCTTGAAG
AAAATATTTCTAGTTCTCTGCAAAATTGCCAACAGCCTTGATCAGTCTGTTGTAGACCTGCCCCAGATGTT
CCCTTGCCTCACAGAGCTCACAATGGATCATTGCGATGATTTATGTGAGCTTCCTTCAAGCATTTCAAGGG
TGCACTCGCTTGAATGCATGAGTATCACCAACTGCCACAGCCTCCAAGAACTGCCAGCCGACCTGGGAAAA
CTGAATTCCCTACAAATTCTTAGGGTTTATGATTGCCCAAGTCTGAAGACGCTTCCTCCTGGCCTATGTGA
ATTGGAGTGTTTGAAGTATCTTGACATTTCTCAGTGTGTTGGTCTGGAATGCCTTCCTGAAGGAATCGGTG
GATTGTTAAAGTTGGAAAAGATCGACATGAGGAAGTGCTCACGGATAAGAAATCTACCAAAATCTGCTGCC
TCATTGCAATTGCTGCGTCATGTGATTTGTGATGAAGAGATTTCTTGGTTGTGGAAAGATGTGGAGACGGC
TGTACCAGGTGTTCATGTGGAGTTTGCAAAGGAATGCTTTGATCTGGACTGGCTGGTGGAGTGACCAAGGT
TTATGACTTGTATTTACTATTGTTGTTGTCCTAAGCAAGAGTAAATATTTTCTTTCATCCAATGTAAATAA
TTTTCCTCTTGGAAATTTAAATGGAAAGGGTGGAGAGTTTAGTGATTACTCCTCCATTCTACTGTATG
>VpRPW8-b ORF 1-2472
ATGGCTTTGGCGCTTGTTGGAGGGGCTGCTCTGGGAGCAGCGTTTCAGGGTTTGCTAACGGCGGTCATAAA
AGTAAGTAAAAAGTTTGCCGGGTTCCACTCCATCCTCAAAAAACTCGAAGCCACACTCGAACGCATAAAGC
CATATATCCAAGAGATGGAAAGGTTGAACGACGAGTTGGATCGTCCAAGGATGGAAATGGAGAAGTTGATC
CAAATCTTACAAGACGGAGAGAAGCTAATCCAAGACTGCTCTCGCTGCTACTGCTACCAGAGGATAGGGTA
CGCCAATAAAATTAAAGCCCTCGATGCCTCTCTTCTTAGATTGTTTCAGGTGGATATGCACGCCCAAGTCA
GTAGGGACGTCGAGGAGATTCTGGCCATTCTCAAATCAAATGGATGCAATTGGAATTACAGAGGGGTTTCC
GATGAACATGAAAACTTGGGTTCCTGCAACGCTCCTGGTCCACCGGAATTTATGGTGGGATTAGATGTGCC
TCTCAAAGAATTGAAGAGGCGGCTATGCGAGGATGGGGAATCAAGGATTGTGATCAGGGCTCCTGGAGGAT
GTGGGAAAACCACTTTGGCTAAAGGGCTTTGTCACGACAATCAAGTCAGAGAATATTTTAAGCACATTTTG
TATGCCACGGTGTCAAGACCGCCCAACCTAATTGCTATCATTACGAAACTATTTTGGGACGAGGATGAACA
AGTGCCAAAGTTTCAAAGTGAGGAAGATGCAGCCAATCAAATGGAACTGAAACTGAACAAGAAAGAAGAAT
CTGGTGCTGTACTGTTGGTCCTAGATGATGTTTGGTGTGGATCAGAATCCCTCTTAGCCAAGTTCAAGTTC
CGGACATCAAAATCCAAGGTCGTCGTTACATCAAGAAATGAATTTCCAGAATTTGGCTCTACATATGACTT
GGAATTGTTGAATGACGACGATGCCATGGCTCTTTTTCGTCACTCAGCAATCCCCCAGAATGGAAGCTGTA
ATTACACACCGACTGATAGGCTTGTGAAGAAGATAGTGGGGCACTGCAAGGGACTTCCACTGGCCCTGGAA

GTCGTTGGCAGATCACTCCATGGGCGGCCTGTAGAGATCTGGAGAAGCCGACTGAAGAAATTATCCGAAGG
TCAATCCATTGTCAATTCTGAAACTGATCTGCGTAAATGTCTTCAAAGTAGCTTAGACGCCCTAAATGACG
AGGATGTTATGCTGAAGGAGTGTTTTATGGACTTGGGCTCCTTTCCTGAAGACCAGGAAATCCCTGCCACT
GCTCTTATAGACATGTGGGCGGAATTGTACAACCTAGATAAAGACGGGGTTCATGCCATTGCGAACCTTCA
CAAACTCTCCTCTCAGACTCTGCTTAATCTTGCGGTCACGAGGAATGATGCAAGTGAGATTGATGGATGGT
ACAGCGATGCCATTGTGATGCAGCATGATCTTCTCAGGGACCTAGCCATTTACGAGAGCAAACAGGAGCTC
ATAAAAGAGAGGAAAAGACTATTTGTGGACTTTACCGGTAGCAAACTCCCAGAGTGGTGGACTGAAAAAGA
GCAACCCCGATCAAGTGCTCGCCTTGTGTCCATCTCTACAGGTGAAATGTTTTCCTCAAGCCAGGGCGACC
TGCAAATTCCTGAAACCGAGGTTCACTGGTGCAACATGCAAATTCCTGACCCTGAGGTTCTAATACTGAAC
TTTAATCAGACACAGAAAAAATACAAATTGCCTGAGTTCATTAAGCAAATGGATAAACTGAAGGTTCTAAT
AGTAACAAATTATGGTATCGCAGTTGAACTGACTAATTTTTCAGTACTCGGCTCCTTGTCCAATCTAAAGA
GAATCAGGTTAGAGAAAGTTTCGATTCCAACACTGTGCAACACCAGTATGGTATTGAAGAATTTGGAGAAG
ATATCCTTAGTCATGTGTTATAAGATTGGTCAGGCTTTTGCAAGTAGTACCATCCAGATTACAGAAATGTT
AGCCAACCTTAGGGAAATCAACATTGACTACTGTAATGACTTGGTGGAATTACCAGAGGGGTTTTGTGATT
TAGTCCGACTGAATAAGCTGAGCATCAGCAACTGCCCTAAGCTGTCTGCACTGCCGGAAGGAATAGGGAAG
CTTGCAAATCTGGAAGTGCTAAGGCTTCGTGCCTGTGCATGGGTGTCAAAATTGCCAGACTCAATTGGAAG
CCTCCACAAGTTGAGCTTTCTTGATATAACTGGTTGTGTACGACTGTCGGAAATGCCGAACCGAATAGGTG
GGTTGCGTGATTTAAGAGAGTTCCACATGAGAAGGTGCCCTGGTTTGTGCGAGCTGCCATCATCAGTGAAG
GATCTCGTGGATTTGGAGAGTGTAATATGCGATGAAAGGACTGTCCTGCTGTGGGAATCTTTTAAGCACTT
CCTCCCCAATCTCACCCTATCAGTGCGTGAAGAAAGTATCAACTGGCATTTGCTATAAAATCCTAGCTTCT
CGAAAAAAAATTCTCCTCAACAGGCGAGGGATTGCAAAATGGAAGCAGCTGTACATGTAAGCATTGG
>VpRPW8-c ORF 1-2448
ATGGCTTTGGAGCTTGTTGGAGGGGCTGCTTTGGGAGCAGTGTTTGAGAAGTTGTTGGCGGCGGTTGTAGA
TGCAAGCAATAAGGCAACTCAGTTCGAGTCCAGCCTCAAAAAACTCGAAGAGACACTCAAATCCATAAATC
CAAGTATCCTAGAGATGAAAAGGATGAACGACCAGTTGGATCGTCCAAAGGAGGACATGGAGAAGTTGATC
CAAATCTTAAAAGATGGGGAGAAGCTAATCCACAAGTGCTCCAAGGTCTCTTGTTGCAGCTACTTCAAGAA
GTGGAGGTACGCCAATAAAATTGAGGCCTTGGAGGACTCTCTTCTTAAATTTTTTCAGGTGGAATTGCAAG
CCCAACTCAGTAGGAACAACATGCAGATTCTGGTCCTGCTCAAATCAAATAGATTCAGTTGGAGTAACAGA
GGGGTTTCCGTTAAATATGAAAGTTTGGGTTCCTGTGAGGCTACTGATCCGCCGGATTTTATGGTGGGACT
AGATGTGCCTCTCAAAGAATTGAAGAGGTGGCTGTTTACGGATGGGGAATCAAGGATTGTGGTGTCTGCTC
CTGGAGGATGTGGGAAAACCACTTTGGCTAAAAGGCTTTGTCACGACCAACAAGTCAAGGAATATTTTCAG
CACATTTTCTATGTCACGGTGTCAAAAACATTCAACCTAATTGGCATCATCAAGAAACTATTTTGGCATAG
TGATGAACAAGTGCCGGGGTTTCAAAATGAGGAAGATGCAGTCAACCAATTGGAACTAATGCTGAAGAGGA
AAGTAGAATCTGGTCGTATACTGTTGGTCCTAGATGACGTTTGGTCTGGGTCGGAATCTTTCCTAACGAAG
TTTAACTTCCAAATATCAGGATGCAAGGTTCTGATTACATCTAGAAATGAATTTCCAAAATTTGGTTCTAC
ATATAACTTGAAATTGTTGAGTGAAGAAGATGCCAAGACTCTTTTCCGTCACTCAGCAATCCCTGAGGATG
GGAGTGGTTCTTCCATGCCCGGTGAAGGCCTTGTGAATACGATAGTGAGGCGCTGCAAGGGATTTCCACTG
GCCCTGGAAGTGGTTGGCAGATCGCTCCATGGGCAGCCTGTAGAGATCTGGAGAAGCACACTGATGAAATT
ATCTGAAGGTGAATCCATTGTCAATTCTGAAGATGAACTGCGTAATTGTCTTCAGAGTAGCTTAGATGCCC
TTGATGACAAGGATATTATGCTGAAGGAGTGTTTTATGGACTTGGGTTCATTTCCTGAAGACCAAAAAATC
CCTGCCACTGCTCTTATAGATATGTGGGCGGAATTGCACAAACTAGATAAAGACGGCATTTATGCCATTTC
CAACCTTCAGAAACCCTGCTCTCGGAATCTGCTTAATCTTGTGGTCACAAGGAATGATGCAAATGAGATTG
ATTGGTGCTACAATGATGCCTTTGTCATGCAGCATGATCTTCTCAGGGATCTAGCCATTTATCAGAGCAAC
CAGGAGCCCATAGAAAAGAGGAAAAGACTAATCATGGACTTGACAGGAAACAGACTCCCCGAGTGGTGGAC
TAAAGAAAATCAACCCCAATTAAGTGCTCGTCTTGTGTCCATCTCCACAGATGAAATGTTCTCCTCAAGCT
GGTGCAACATGCAACTTCCTGAAGCTGAGGCTCTAGTACTGAACTTCAATCAGACAGAAAATAAATACGAA
TTGCCAGAGTTCATGAAGCAAATGGATAAACTGAAGGTTCTAGTAGTAACAAATTATGGTTTCTGTGCTGC
TGAATTGACTAATTTTTCAGTACTTGGTTCCTTATCCAATCTAAAGAGAATCAGGTTAGAGCAAGTTTCAA
TTCCAACACTATGCAACACGAGTATGGAATTGAAGAATCTGGAAAAGCTATCCTTAGTCATGTGTCATAAG
ATTGGTCAGGCTTTTGCAAGTAGTACCATCCAGATCCCAGAAATGTTACCAAACCTTAGGGAAATCAACAT
TGATTACTGTAATGACTTGGTGGAATTACCAGAAGGGTTTTGCGACTTAGTCCAGCTGAATAAGCTGAGCA
TCAGCAACTGCCATAAGCTGTCTGCACTGCCGGAAGGGATAGGGAAGCTTGCAAATCTGGACGTGCTAAGG
GTTAGTGCCTGTACATTGGTGTCAAAATTGCCAGACTCAATGGGAAGCCTCCACAAGTTGAGGGTTCTTGA
TATAACTGGTTGTTTACGAGTAAGGAAAATGCCGAAACAAATAGGGGAGTTGCGTGGTCTAAGAGAGCTCC
ACATGAGAAGGTGCCCAGGTTTGCGCGAGCTGCCACCATCAGTGACGCTTCTCGTGGATTTGGAGAGGGTA
ATCTGCGATGAAGAGACTGCCCAGCTGTGGGAATGTTTTACGCACTTGCTCCCCAATCTCACCCTATCAGT
GCCTGAAGAAATTATCAGCTTGAATTGGCTTTAAAATACTGGTTTCTGAAAAACTTTCATCCTCCAAAGTG

CTGAACCGGACGCCTAATTATATTC
>VpRPW8-d ORF 1-1944
ATGGCTTTGGAGCTTGTTGGAGGGGCTGCTTTGGGAGCAGTGTTTGAGAAGTTGTTGGCGGCGGTTGTAGA
TGCAAGCAATAAGGCAACTCAGTTCGAGTCCAGCCTCAAAAAACTCGAAGAGACACTCAAATCCATAAATC
CAAGTATCCTAGAGATGAAAAGGATGAACGACCAGTTGGATCGTCCAAAGGAGGACATGGAGAAGTTGATC
CAAATCTTAAAAGATGGGGAGAAGCTAATCCACAAGTGCTCCAAGGTCTCTTGTTGCAGCTACTTCAAGAA
GTGGAGGTACGCCAATAAAATTGAGGCCTTGGAGGACTCTCTTCTTAAATTTTTTCAGGTGGAATTGCAAG
CCCAACTCAGTAGGAACAACATGCAGATTCTGGTCCTGCTCAAATCAAATAGATTCAGTTGGAGTAACAGA
GGGGTTTCCGTTAAATATGAAAGTTTGGGTTCCTGTGAGGCTACTGATCCGCCGGATTTTATGGTGGGACT
AGATGTGCCTCTCAAAGAATTGAAGAGGTGGCTGTTTACGGATGGGGAATCAAGGATTGTGGTGTCTGCTC
CTGGAGGATGTGGGAAAACCACTTTGGCTAAAAGGCTTTGTCACGACCAACAAGTCAAGGAATATTTTCAG
CACATTTTCTATGTCACGGTGTCAAAAACATTCAACCTAATTGGCATCATCAAGAAACTATTTTGGCATAG
TGATGAACAAGTGCCGGGGTTTCAAAATGAGGAAGATGCAGTCAACCAATTGGAACTAATGCTGAAGAGGA
AAGTAGAATCTGGTCGTATACTGTTGGTCCTAGATGACGTTTGGTCTGGGTCGGAATCTTTCCTAACGAAG
TTTAACTTCCAAATATCAGGATGCAAGGTTCTGATTACATCTAGAAATGAATTTCCAAAATTTGGTTCTAC
ATATAACTTGAAATTGTTGAGTGAAGAAGATGCCAAGACTCTTTTCCGTCATTCAGCAATCCCTGAGGATG
GGAGTGGTTCTTCCATGCCCGGTGAAGACCTTGTGAATACGATAGTGAGGCGCTGCAAGGGATTTCCACTG
GCCCTGGAAGTGGTTGGCAGATCGCTCCATGGGCAGCCTGTAGAGATCTGGAGAAGCACACTGATGAAATT
ATCTGAAGGTGAATCCATTGTCAATTCTGAAGATGAACTGCGTAATTGTCTTCAGAGTAGCTTAGATGCCC
TTGATGACAAGGATATTATGCTGAAGGAGTGTTTTATGGACTTGGGTTCATTTCCTGAAGACCAAAAAATC
CCTGCCACTGCTCTTATAGATATGTGGGCGGAATTGCACAAACTAGATAAAGACGGCATTTATGCCATTTC
CAACCTTCAGAAACTCTGCTCTCGGAATCTGCTTAATCTTGTGGTCACAAGGAATGATGCAAATGAGATTG
ATTGGTGCTACAATGATGCCTTTGTCATGCAGCATGATCTTCTCAGGGATCTAGCCATTTATCAGAGCAAC
CAGGAGCCCATAGAAAAGAGGAAAAGACTAATCATGGACTTGACAGGAAACAGACTCCCCGAGTGGTGGAC
TAAAGAAAATCAACCCCAATTAAGTGCTCGTCTTGTGTCCATCTCCACAGATGAAATGTTCTCCTCAAGCT
GGTGCAACATGCAACTTCCTGAAGCTGAGGCTCTAGTACTGAACTTCAATCAGACAGAAAATAAATACGAA
TTGCCAGAGTTCATGAAGCAAATGGATAAACTGAAGGTTCTAGTAGTAACAAATTATGGTTTCTGTGCTGC
TGAATTGACTAATTTTTCAGTACTTGGTTCCTTATCCAATCTAAAGAGAATCAGGTTAGAGCAAGTTTCAA
TTCCAACACTATGCAACACGAGTATGGAATTGAAGAATCTGGAAAAGCTATCCTTAGTCATGTGTCATAAG
ATTGGTCAAGCTTTTGCAACTGAATAA
>VpRPW8-e ORF 1-2448
ATGTCTGCGGAGCTTGTTGGAGGGTCTGCTTTGGGAGCAGTGTTTGAGAAGTTGTTCGCGGCGGTTGTAGA
TGCAAGCAATAAGGCAACTCAGTTCGAGTCCAGCCTCAAAAAACTCGAAGAGACACTCAAATCCATAAATC
CAAGTGTCCTAGAGATGAAAAGGATGAACGATCAGTTGGATCGTCCAAAGGAGGACATGGAGAAGTTGATC
CAAATCTTAAAAGATGGGGAGAAGCTAATCCACAAGTGCTCCAAGGTCTCTTGTTGCAGCTACTTCAAGAA
GTGGAGGTACGCCAATAAAATTGAGGCCTTGGAGGACTCTCTTCTTAAATTTTTTCAGGTGGAATTGCAAG
CCCAACTCAGTAGGAACAACATGCAGATTCTGGTCCTGCTCAAATCAAATAGATTCAGTTGGAGTAACAGA
GGGGTTTCCGTTAAATATGAAAGTTTGGGTTCCTGTGAGGCTACTGATCCGCCGGATTTTATGGTGGGACT
AGATGTGCCTCTCAAAGAATTGAAGAGGTGGCTGTTTACGGATGGGGAATCAAGGATTGTGGTGTCTGCTC
CTGGAGGATGTGGGAAAACCACTTTGGCTAAAAGGCTTTGTCACGACCAACAAGTCAAGGAATATTTTCAA
CACATTTTCTATGTCACGGTGTCAAAAACATTCAACCTAATTGGCATCATCAAGAAACTATTTTGGCATAG
TGATGAACAAGTGCCGGGGTTTCAAAATGAGGAAGATGCAGTCAACCAATTGGAACTAATGCTGAAGAGGA
AAGTAGAATCTGGTCGTATACTGTTGGTCCTAGATGACGTTTGGTCTGGGTCGGAATCTTTCCTAACGAAG
TTTAACTTCCAAATATCAGGATGCAAGGTTCTGATTACATCTAGAAATGAATTTCCAAAATTTGGTTCTAC
ATATAACTTGAAATTGTTGAGTGAAGAAGATGCCAAGACTCTTTTCCGTCACTCAGCAATCCCTGAGGATG
GGAGTGGTTCTTCCATGCCCGGTGAAGACCTTGTGAATACGATAGTGAGGCGCTGCAAGGGATTTCCACTG
GCCCTGGAAGTGGTTGGCAGATCGCTCCATGGGCAGCCTGTAGAGATCTGGAGAAGCACACTGATGAAATT
ATCTGAAGGTGAATCCATTGTCAATTCTGAAGATGTACTGCGTAATTGTCTTCAGAGTAGCTTAGATGCCC
TTGATGACAAGGATATTATGCTGAAGGAGTGTTTTATGGACTTGGGTTCATTTCCTGAAGACCAAAAAATC
CCTGCCACTGCTCTTATAGATATGTGGGCAGAATTGCACAAACTAGATAAAGACGGCATTTATGCCATTTC
CAACCTTCAGAAACTCTGCTCTCGGAATCTGCTTAATCTTGTGGTCACAAGGAATGATGCAAATGAGATTG
ATTGGTGCTACAATGATGCCTTTGTCATGCAGCATGATCTTCTCAGGGATCTAGCCATTTATCAGAGCAAC
CAGGAGCCCATAGAAAAGAGGAAAAGGCTAATCATGGACTTGACAGGAAACAGACTCCCCNAGTGGTGGAC
TAAAGAAAATCAACCCCAATTAAGTGCTCGTCTTGTGTCCATCTCCACAGATGAAATGTTCTCCTCAAGCT
GGTGCAACATGCAACTTCCTGAAGCTGAGGCTCTAATACTGAACTTCAATCAGACAGAAAATAAATACGAA
TTGCCAGAGTTCATGAAGCAAATGGATAAACTGAAGGTTCTAGTAGTAACAAATTATGGTTTCTGTGCTGC
TGAATTGACTAATTTTTCAGTACTTGGTTCCTTATCCAATCTAAAGAGAATCGGGTTAGAGCAAGTTTCAA

TTCCAACACTATGCAACACGAGTATGGAATTGAAGAATCTGGAAAAGCTATCCTTAGTCATGTGTCATAAG
ATTGGTCAGGCTTTTGCAAGTAGTACCATCCAGATCCCAGAAATGTTACCAAACCTTAGGGAAATCAACAT
TGATTACTGTAATGACTTGGTGGAATTACCAGAAGGGTTTTGTGACTTAGTCCAGCTGAATAAGCTGAGCA
TCAGCAACTGCCATAAGCTGTCTGCACTGCCGGAAGGGATAGGGAAGCTTGCAAATCTGGAAGTGCTAAGG
GTTAGTGCCTGTACATTGGTGTCAAAATTGCCAGAATCAATAGGAAACCTCCACAGGTTGAGGGTTCTTGA
TATAACTGGTTGTTTACGAATAAGGAAAATGCCGAAACAAATAGGGGAGTTGCGTGGTCTAAGAGAGCTCC
ACATGAGAAGGTGCCCAGGTTTGCGCGAGCTGCCACCATCAGTGACGCTTCTCGTGGATTTGGAGAGGGTA
ATCTGCGATGAAGAGACTGCCCAGCTGTGGGAATGTTATACGCACTTGCTCCCCAATCTCACCCTATCAGT
GCCTGAAGAAATTATCAACTTGAATTGGCTTTAA
